# Supplementary material for: Causal Relationship between Aging and Anorexia Nervosa: A White-Matter-Microstructure-Mediated Mendelian Randomization Analysis
Source: Biomedicines. 2024 Aug 16;12(8):1874. doi: 10.3390/biomedicines12081874 (PMC11351342; doi:10.3390/biomedicines12081874)
Supplement: Supplementary file 1 [file biomedicines-12-01874-s001.zip › Supplementary Figure/Figure S4/Singe SNP plot of MR analysis for TL to ubm-b-2040.pdf]

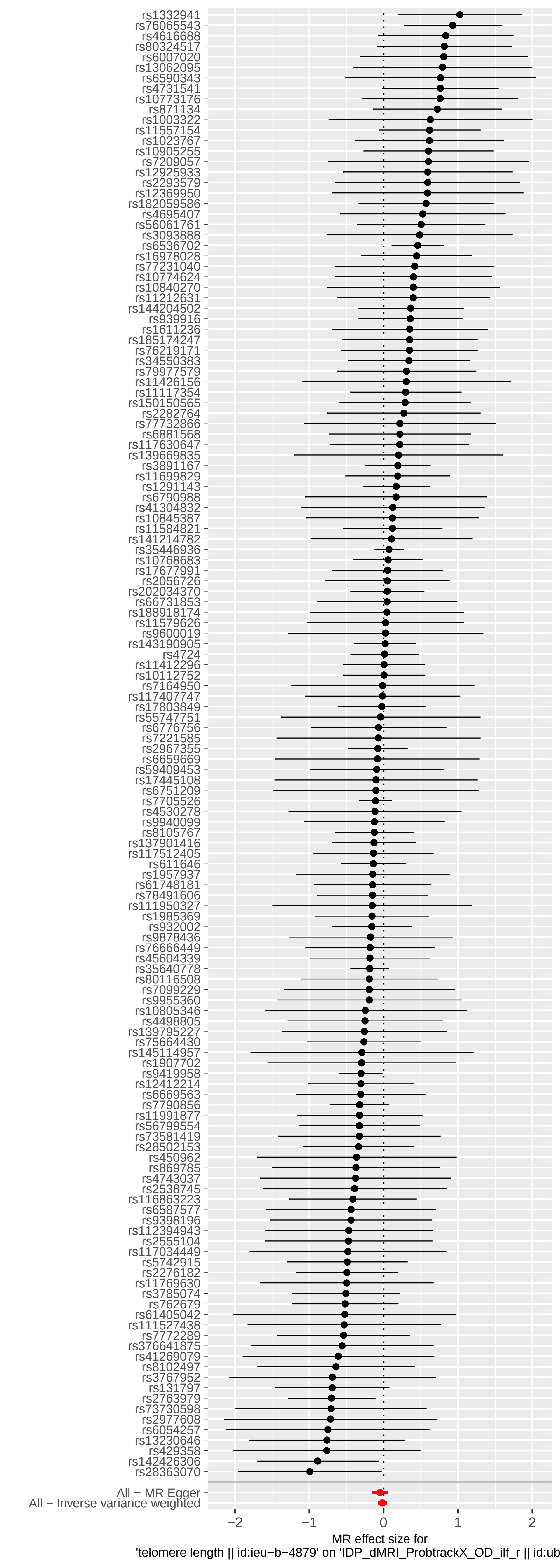
rs1332941
rs76065543
rs4616688
rs80324517
rs6007020
rs13062095
rs6590343
rs4731541
rs10773176
rs871134
rs1003322
rs11557154
rs1023767
rs10905255
rs7209057
rs12925933
rs2293579
rs12369950
rs182059586
rs4695407
rs56061761
rs3093888
rs6536702
rs16978028
rs77231040
rs10774624
rs10840270
rs11212631
rs144204502
rs939916
rs1611236
rs185174247
rs76219171
rs34550383
rs79977579
rs11426156
rs11117354
rs150150565
rs2282764
rs77732866
rs6881568
rs117630647
rs139669835
rs3891167
rs11699829
rs1291143
rs6790988
rs41304832
rs10845387
rs11584821
rs141214782
rs35446936
rs10768683
rs17677991
rs2056726
rs202034370
rs66731853
rs188918174
rs11579626
rs9600019
rs143190905
rs4724
rs11412296
rs10112752
rs7164950
rs117407747
rs17803849
rs55747751
rs6776756
rs7221585
rs2967355
rs6659669
rs59409453
rs17445108
rs6751209
rs7705526
rs4530278
rs9940099
rs8105767
rs137901416
rs117512405
rs611646
rs1957937
rs61748181
rs78491606
rs111950327
rs1985369
rs932002
rs9878436
rs76666449
rs45604339
rs35640778
rs80116508
rs7099229
rs9955360
rs10805346
rs4498805
rs139795227
rs75664430
rs145114957
rs1907702
rs9419958
rs12412214
rs6669563
rs7790856
rs11991877
rs56799554
rs73581419
rs28502153
rs450962
rs869785
rs4743037
rs2538745
rs116863223
rs6587577
rs9398196
rs112394943
rs2555104
rs117034449
rs5742915
rs2276182
rs11769630
rs3785074
rs762679
rs61405042
rs111527438
rs7772289
rs376641875
rs41269079
rs8102497
rs3767952
rs131797
rs2763979
rs73730598
rs2977608
rs6054257
rs13230646
rs429358
rs142426306
rs28363070
All − MR Egger
All − Inverse variance weighted
−2
−1
0
1
2
MR effect size for
'telomere length || id:ieu−b−4879' on 'IDP_dMRI_ProbtrackX_OD_ilf_r || id:ub
